# A mixed methods study of an online intervention to reduce perfectionism

**DOI:** 10.1007/s12144-022-02953-y

**Published:** 2022-04-04

**Authors:** Shanara Visvalingam, Hannah L. McHardy, Susanne J. Norder, Natasha R. Magson, Melissa M. Norberg

**Affiliations:** Centre for Emotional Health, School of Psychological Sciences, Macquarie University, Building 4 First Walk, Room 714, Sydney, NSW 2109 Australia

**Keywords:** Perfectionism, Mindfulness, Empathy, Brief psychotherapy, Internet-based therapy

## Abstract

Perfectionism is linked to a variety of mental health conditions in university students. Guided by the Perfectionism Social Disconnection Model, the purpose of the current mixed methods feasibility study was to evaluate the acceptability and potential effectiveness of a brief online intervention designed to reduce the negative consequences of perfectionism in university students. Seventy university students (83.9% female; *M*_*age*_ = 19) reporting moderate to extreme levels of perfectionism completed the two hour ‘Intentional Imperfection Program’ (IIP). The IIP includes techniques to increase mindfulness, compassion for self and others, distress tolerance, and social skills. Participants completed self-report measures at baseline and at a two-week follow-up. Quantitative data showed statistically significant small to moderate reductions in self-oriented perfectionism (*d* = −0.48, *p* < .001), socially-prescribed perfectionism (*d* = 0.40, *p* < .001), hostility (*r* = 0.53, *p* < .001), rejection sensitivity (*d* = 0.37, *p* < .001), depression (*r* = −0.47, *p* < .001), and anxiety (*r* = −0.33, *p* = .010) and a small increase in perceived social support (*r* = −0.29, *p* = .023). Thematic analyses of qualitative data indicated that participants found the IIP feasible, enjoyable, and useful. A brief online intervention may be a feasible way of reducing the negative consequences of perfectionism among university students. A randomised control trial is warranted to further evaluate the efficacy of the IIP. This research was registered with the Australian New Zealand Clinical Trials Registry (no. ACTRN12620000574943).

## A Mixed Methods Study of an Online Intervention to Reduce Perfectionism

Perfectionism is characterised by the pursuit of high standards accompanied by critical evaluation of the self and others (Hewitt et al., 2017). Hewitt and colleagues (1991) described three facets of perfectionism: socially-prescribed perfectionism (i.e., belief that others expect them to be perfect), self-oriented perfectionism (i.e., tendency to set high standards for oneself) and other-oriented perfectionism (i.e., having unrealistically high standards of others). Prior research with university samples have found all three facets of perfectionism to predict psychological distress (Schweitzer & Hamilton, 2002) and poor academic performance (Rice et al., 2016), suggesting that perfectionism confers risk for poor psychological well-being in university students. Considering that one in three university students meet criteria for a psychological disorder (Lipson et al., 2019) and that perfectionism is a significant problem for university students, with prevalence estimates as high as 25% (Curran & Hill, 2017), programs that target perfectionism may reduce students’ vulnerability to psychopathology and improve their academic outcomes.

## Perfectionism Social Disconnection Model

The Perfectionism Social Disconnection Model (PSDM) describes two pathways that connect perfectionistic traits to psychopathology and poor psychological well-being (Hewitt et al., 2017). The first pathway links perfectionism to off-putting hostile interpersonal behaviours, such as inappropriate social responses (e.g., overt or subtle aggression, coldness, aloofness; Hewitt et al., 2017). The second pathway links perfectionism to rejection sensitivity, the cognitive process whereby individuals readily anticipate rejection, interpret the behaviours of others as indicative of lack of acceptance, and perceive the judgments of others as critical (Hewitt et al., 2017). According to the PDSM, both hostile interpersonal behaviours and rejection sensitivity lead to perceived or actual social disconnection, which in turn, leads to poor psychological well-being (Hewitt et al., 2017).

Increasingly, research has supported the links specified in the PSDM. For instance, research has found a link between the three facets of perfectionism and rejection sensitivity, with the strongest associations found for socially-prescribed perfectionism (Flett et al., 2014). In addition, the three facets of perfectionism have been linked to hostile interpersonal behaviours, with the strongest links found for other-oriented and socially-prescribed perfectionism (Stoeber et al., 2017). Moreover, longitudinal studies have shown that perceived and actual social disconnection mediates the link between perfectionism and depressive symptoms (Cha, 2016; Mackinnon et al., 2017). Thus, techniques that target rejection sensitivity, interpersonal hostility, and social disconnection may reduce the harmful consequences associated with perfectionism.

## Approaches to Treatment

Mindfulness (i.e., the ability to approach situations with nonjudgement, nonreactivity, and awareness) and increased self-compassion (i.e., fostering a sense of empathy for oneself) may help to manage and reduce rejection sensitivity (Abdollahi et al., 2020; Sakiz & Saricam, 2015). Research suggests that dispositional mindfulness is associated with lower rejection sensitivity (Hafner et al., 2018) and that mindful meditation may be effective in reducing emotional reactivity and anxiety following social rejection (Joss et al., 2020). In addition, self-compassion has been shown to moderate the link between perfectionism and depression such that greater self-compassion reduces depressive symptoms (Abdollahi et al., 2020). Further, greater self-compassion also predicts lower levels of rejection sensitive behaviour (Sakiz & Saricam, 2015). Thus, addressing the cognitive faculties of mindfulness and self-compassion may reduce sensitivity to rejection and negative affect.

Increasing one’s empathy for others and tolerance for distress may help to manage interpersonal hostility. Some studies have shown that mindfulness-based compassion meditations focused on fostering empathy for others can reduce interpersonally hostile behaviours and increase interpersonal relations (Shonin et al., 2015). Some studies also have shown that an increased capacity for distress tolerance (i.e., the capability to experience and endure negative emotional states) predicts lower levels of hostility and anger (Matheny et al., 2017), such that emotional exposure and behavioural practice can increase tolerance for distress and reduce depression and anxiety symptoms (Wright et al., 2020). Thus, engaging in mindfulness-based compassion meditations and activities to increase distress tolerance may help to reduce interpersonal hostility.

Although mindfulness, compassion for the self and others, and distress tolerance may reduce rejection sensitivity and interpersonal hostility, which should in turn reduce social disconnection, technique(s) that directly target social connectedness may also be helpful. Social skills training is one strategy that has previously demonstrated efficacy in improving social competence and self-efficacy to increase social connectedness (Spence, 2003). Social skills training may include psychoeducation about and the observation and initiating of fostering friendships, initiating conversations, engaging in active listening, and being assertive (Spence, 2003). In addition, studies have shown that friendship goal setting has been associated with increased social support and lower dropout rates in university students (Kim & Kim, 2011). Thus, social skills training, in addition to goal setting, may help to decrease social disconnection and increase social connectedness.

## Rationale for the Study

Previous research suggests that university students rarely seek or receive psychological treatment, often citing time and financial constraints as the main barriers to receiving treatment (e.g., Czyz et al., 2013). Existing perfectionism interventions (cognitive behavioural and psychodynamic) often involve eight or more 1-h sessions delivered weekly or bi-weekly (Hewitt et al., 2015; Lloyd et al., 2015; Suh et al., 2019), which may hinder time-poor and financially strapped university students from accessing them. As such, there appears to be a need for a brief, effective, and affordable intervention that targets the negative consequences associated with perfectionism among university students.

To directly address this need, this study reports on the development and feasibility of the Intentional Imperfection Program (IIP), a brief intervention aimed at helping university students manage their perfectionism. As previous research suggests that web-based interventions are most beneficial to university students experiencing difficulties rather than all students (Coudray et al., 2019), we tailored our program to fit university students experiencing problematic perfectionistic tendencies. We expected that participation in the IIP, which includes techniques to increase mindfulness, compassion (for self and others), distress tolerance, and social skills, would decrease rejection sensitivity, hostility, depression, and anxiety, and increase perceived and actual social support for university students experiencing perfectionism. We also expected these university students to find the IIP, feasible, enjoyable, and useful.

## Method

### Participants

**Fig. 1:**
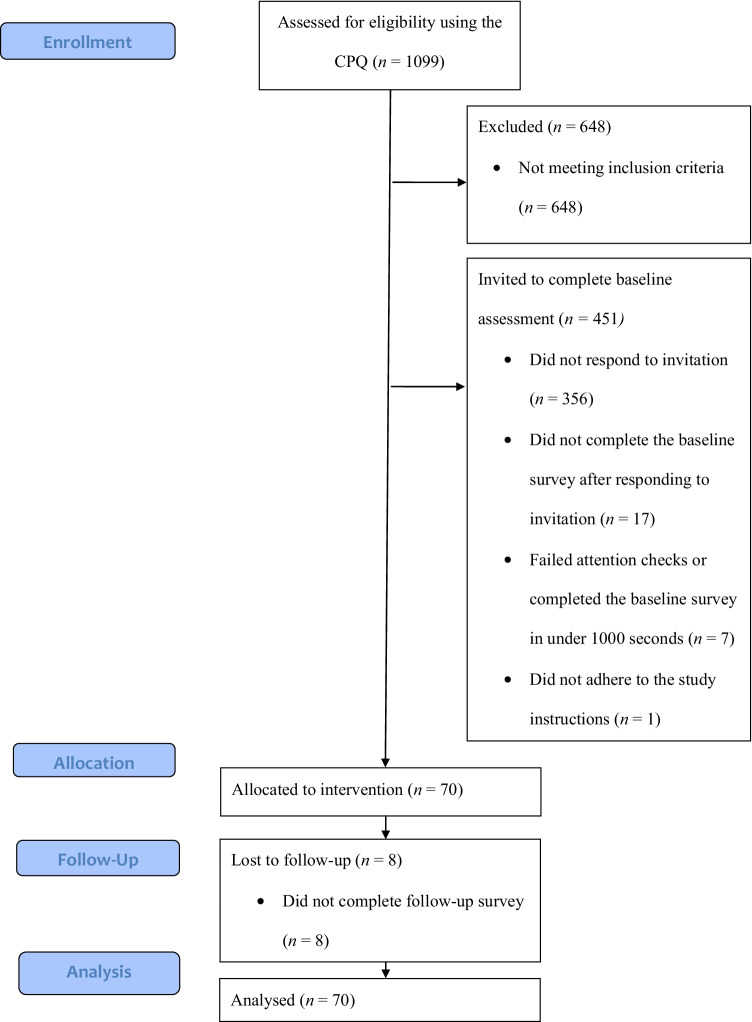
A flowchart depicting participant flow in the study

Four hundred fifty-one first-year psychology students scoring 36 or above on the Clinical Perfectionism Questionnaire (CPQ; Fairburn et al., 2003) were invited to participate in this study and 70 participated after completing the baseline assessment (see Fig. 1 for CONSORT flowchart). There were no exclusion criteria. A priori power analyses indicated that 53 participants would be needed to detect small pre-post differences (90% power, α = 0.05). Table 1 displays included participants’ demographic and clinical characteristics.

**Table 1:** Demographic and clinical characteristics by group with comparison analyses

	Total		Completers		Dropouts		Comparison Statistics	Effect size
	(*n* = 70)		(*n* = 62)		(*n* = 8)			
	*M*	*SD*	*M*	*SD*	*M*	*SD*	*t or U*	*d or r*
Age (*Mdn, IQR*)	19	18–20	19	18–20	19	18–19	240.00	−0.01
CPQ	38.53	2.12	38.63	2.19	37.75	1.39	−1.103	0.47
HF MPS-45								
Socially-prescribed	56.80	14.76	55.40	14.21	67.63	15.26	2.270*	0.82
Self-oriented	78.73	13.77	79.51	13.59	72.62	14.45	−1.340	0.49
Other-oriented	53.76	12.62	54.08	12.37	51.25	15.15	−0.594	0.20
	*n*	*%*	*n*	*%*	*n*	*%*	*χ*^*2*^	*φ* or *V*
Gender							1.51	0.14
Female	60	60.0	52	83.9	8	100.0		
Male	9	9.0	9	14.5	0	0.0		
Transgender/Intersex	1	1.0	1	1.6	0	0.0		
Ethnicity							5.16	0.27
Anglo Australian	36	51.4	32	51.6	4	50.0		
European	8	11.4	7	11.3	1	12.5		
Middle Eastern	5	7.2	3	4.8	2	25.0		
Asian	15	21.4	14	22.6	1	12.5		
Other	6	8.6	6	9.7	0	0.0		
Relationship Status							0.70	0.10
Single	41	58.6	36	58.1	5	62.5		
In a relationship	24	34.3	21	33.9	3	37.5		
Married	5	7.1	5	8.1	0	0.0		
Employment Status							5.09*	−0.27
Employed	50	71.4	47	75.8	3	37.5		
Unemployed	20	28.6	15	24.2	5	62.5		
Weekly Income							2.94	0.20
AUD $0 - $300	26	37.1	22	35.5	4	50.0		
AUD $301 - $600	26	37.1	23	37.1	3	37.5		
AUD $601 - $900	10	14.3	10	16.1	0	0.0		
AUS $901 or more	4	5.7	4	6.4	0	0.0		
Prefer not to answer	4	5.7	3	4.8	1	12.5		
Receiving Psychological Treatment	20	28.6	18	29.0	2	25.0	0.05	−0.03
Receiving Psychotropic Medication	13	18.6	11	17.7	2	25.0	0.25	0.06

*p < .05. CPQ = Clinical Perfectionism Questionnaire. HF MPS-45 = Hewitt and Flett Multidimensional Perfectionism Scale-45

### Measures

*Hewitt and Flett Multidimensional Perfectionism Scale (HF MPS-45;* Hewitt et al., 1991*)* has 45 items measuring three facets of perfectionism: self-oriented perfectionism, socially-prescribed perfectionism, and other-oriented perfectionism. The HF-MPS-45 has been previously used in university samples (self-oriented: *M* = 65.27, *SD* = 14.01, socially-prescribed: *M* = 48.17, *SD* = 12.88, other-oriented: *M* = 53.38, *SD* = 12.55) and psychiatric samples (self-oriented: *M* = 69.90, *SD* = 18.03, socially-prescribed: *M* = 58.18, *SD* = 15.53, other-oriented: *M* = 55.23, *SD* = 13.45; Hewitt & Flett, 1991). The HF MPS-45 has previously demonstrated good to acceptable internal consistency and test-retest retest reliability (Hewitt et al., 1991). The current study showed acceptable to excellent internal consistency at baseline (BL) and follow-up (FU; self-oriented: BL α = .88, FU α = .90; socially-prescribed: BL α = .85, FU α = .85; other-oriented: BL α = .76, FU α = .82).

*Clinical Perfectionism Questionnaire (CPQ;* Fairburn et al., 2003*)* has 12 items measuring ‘clinical perfectionism’. We deemed the CPQ to be a relevant, brief screener because previous studies have shown significant positive correlations between the CPQ and the three subscales of the HFMPS (self-oriented perfectionism: *r* = .49; other-oriented perfectionism: *r* = .28; socially-prescribed perfectionism: *r* = .51; Chang & Sanna, 2012) and because two prior studies have used it to screen for clinically significant perfectionism in conjunction with a mental health diagnosis (Glover et al., 2007; Riley et al., 2007). In these two studies, the average CPQ score was 35.52 (Riley et al., 2007) and 33.67 (Glover et al., 2007). Response options on the CPQ range from 1 to 4. A score of 36 is indicative of participants endorsing each item on CPQ as “*sometimes*”. Although this score seems like perfectionism may not be a problem for an individual, the above two studies suggest such a score may be indicative of an Axis I disorder. Hence, participants were required to score 36 or above to be enrolled in this study. The CPQ has also been previously used in university samples (*M* = 26.53, *SD* = 4.76; Chang & Sanna, 2012). The CPQ has previously shown acceptable internal consistency and test-retest reliability (Dickie et al., 2012). In the current study, the scale’s internal consistency was α = .76.

*Rejection Sensitivity Questionnaire (RSQ;* Downey & Feldman*,* 1996*)* is a 36-item scale measuring rejection sensitivity by determining the individual’s response to eighteen hypothetical scenarios. The RSQ has been previously used in an unrestricted university sample (*M* = 9.16, *SD* = 2.99; Ayduk et al., 2008). This measure has previously shown good internal consistency and test-retest reliability (Downey & Feldman, 1996), and the internal consistency in the current study was good at baseline (α = .87) and excellent at follow-up (α = .93).

*Brief Symptom Inventory (BSI-53;* Derogatis & Melisaratos*,* 1983*)* is a 53-item self-report measure that assesses the presence of symptoms on nine psychological dimensions over the past seven days. Only the depression, anxiety, and hostility subscales were used in the current research. The BSI-53 has been previously used in a community sample (depression: *M* = 0.42, *SD* = 0.65, anxiety: *M* = 0.45, *SD* = 0.60, hostility: *M* = 0.44, *SD* = 0.60) and outpatient sample (depression: *M* = 1.99, *SD* = 1.10, anxiety: *M* = 1.87, *SD* = 1.03, hostility: *M* = 1.39, *SD* = 1.02; Ryan, 2007). Previous research has also shown that the BSI-53 is sensitive to intervention-related change (Crameri et al., 2016), and the depression, anxiety, and hostility subscales to have acceptable internal consistency and test re-test reliability (Derogatis & Melisaratos, 1983). In the current study the measure showed good internal consistency at baseline and follow-up (depression: BL α = .87, FU α = .89; anxiety: BL α = .87, α = .89; hostility: BL α = .81, FU α = .82).

*Multidimensional Scale of Perceived Social Support (MSPSS;* Zimet et al., 1988*)* is a 12-item self-report measure that assesses an individual’s perceived social support from family, friends, and significant others. The MSPSS has been previously used in an unrestricted university sample (*M* = 5.81, *SD* = 0.79; Kazarian & McCabe, 1991). The MSPSS has previously demonstrated good internal consistency (α = .88) and test-retest reliability (*r* = .85; Zimet et al., 1988). In the current study, the MSPSS demonstrated excellent internal consistency in the current study at baseline (α = .92) and follow-up (α = .91).

*Inventory of Socially Supportive Behaviours (ISSB;* Barrera et al., 1981*)* is a 40-item measure of objective social support, assessing the frequency and type of prosocial behaviours. The ISSB has been previously used in an unrestricted university sample (*M* = 3.13, *SD* = 0.64; Reyes et al., 2020). The measure has previously produced excellent internal consistency (Mazer & Thompson, 2011) and test-retest reliability (Gottlieb & Bergen, 2010). The measure in the current study showed excellent internal consistency at baseline (α = .94) and at follow-up (α = .97).

#### Demographics

Participants provided information relating to their age, gender, marital status, income, employment, use of psychotropic medication and psychological treatment.

#### Feedback Survey

This survey included a combination of Likert-style questions and open-ended questions. Participants provided information relating to the content, structure, and readability of the program. Participants also reported on their completion of the assigned homework tasks and practice of skills in the last two weeks.

## The Intentional Imperfection Program (IIP)

Detailed information on the administration and content of the IIP can be found in Appendix A. Briefly, this online program was developed by the first, second and last authors, and unless otherwise mentioned, the materials and resources within the program are original and developed specifically for the current research. There were five learning modules structured to address the specific components of the PDSM and each was accompanied by a corresponding work sheet. The learning modules were as follows: (1) psychoeducation on perfectionism, (2) interpersonal sensitivity psychoeducation and management skills, (3) interpersonal hostility psychoeducation and management skills, (4) social disconnection psychoeducation and strategies to increase social connection, and (5) a summary of the learnt materials. The program took approximately two hours to complete and was delivered via PowToon videos which were uploaded to an online e-therapy platform. The program also contained a workbook and homework sheets.

Each module began with a short video providing descriptive information about the construct of interest (e.g., types of perfectionism, characteristics of interpersonal sensitivity), along with common examples of each. Management skills taught within the modules included mindfulness, self-compassion, meditation, distress tolerance, and social skills training. After completing the IIP, participants were assigned daily homework tasks that facilitated practice of the techniques taught by the program.

## Procedure

The study received ethical approval from the university’s Human Research and Ethics Committee and all participants were treated in accordance with the ethical guidelines set out in the National Statement on Ethical Conduct in Human Research (National Health and Medical Research Council, 2007). The research was registered with the Australian New Zealand Clinical Trials Registry (no. ACTRN12620000574943).

Data were collected using rolling recruitment whereby participants were enrolled into the study at different times during the academic year (July to October 2020). Participants were invited by email to participate in exchange for course credit. After providing consent, participants were sent an email which contained their identification number, a link to the Qualtrics survey (i.e., baseline measures) and instructions on how to access the IIP. After participants completed the baseline survey online, they were provided with a link to complete the IIP. Participants were instructed to complete the IIP within 24 h of receiving the link. Two weeks later participants were sent an automated email containing the link to the Qualtrics follow-up survey and a PDF of the program outline. The follow-up survey included the same measures administered at baseline in addition to a program feedback survey. After completing this, participants were sent a debriefing form.

## Statistical Analysis Plan

Prior to testing the main hypotheses, data were first screened for outliers, normality and missingness by obtaining descriptive statistics in SPSSv25. A series of ANOVAs were conducted to compare demographic and clinical characteristics between completers and dropouts. Comparisons were made using independent samples t-tests for numeric variables and chi-square tests for categorical variables. A series of one-sample t-tests were also conducted to compare the baseline characteristics of our sample to characteristics of unrestricted university samples and psychiatric samples from previous studies (Ayduk et al., 2008; Hewitt & Flett, 1991; Kazarian & McCabe, 1991; Reyes et al., 2020; Ryan, 2007). Next, paired samples t-tests were used to evaluate the efficacy of the intervention. When variables were not normally distributed, the corresponding non-parametric alternative was used instead (e.g., Mann-Whitney *U* test; see results for details). Participant dropout in the follow-up survey was then examined to determine whether the data were Missing Completely at Random (MCAR) using Little’s MCAR test. As the data were not MCAR, we handled the data as recommended by Dong and Peng (2013), using maximum-likelihood estimation with an expectation-maximisation algorithm to estimate the missing values. A sensitivity analyses was carried out whereby these analyses were conducted on the sample with the expectation-maximisation algorithm applied (*n* = 70) and not applied (*n* = 62). As the statistical significance did not differ for any variables except for objective social support (details presented in the results below), the analyses with the expectation-maximisation algorithm applied are reported below. The results of the completer analysis can be found in Appendix Table 4.

We evaluated statistical significance in conjunction with effect sizes, as *p*-values alone may be unreliable indicators of effects. As a measure of effect sizes, we reported Pearson’s *r* (0.2, 0.5, and 0.8 represent small, medium, and large effect sizes, respectively) for non-parametric analyses (e.g., Mann Whitney U tests), Cohen’s *d* (0.41, 1.15, and 2.70 represent small, medium, and large effect sizes, respectively) for parametric analyses and Phi and Cramer’s *V* for chi-square tests (Ferguson, 2009).

Lastly, we analysed participant feedback through an open-ended questionnaire to gain an understanding of participant experience of the intervention and to examine the overall acceptability of the IIP. Quantitative feedback data was examined using both descriptive analyses for continuous variables and frequency analyses for categorical variables. Qualitative feedback data was coded in Nvivo 12 by two independent coders (i.e., first and second author) using the thematic and deductive approach described by Braun and Clarke (2006). The resulting overall Cohen’s kappa was 0.96 indicating excellent agreement between raters.

## Results

### Participant Characteristics

Table 1 displays the demographic characteristics of the total sample, stratified by completers and dropouts, and the comparison analyses of participants who completed the follow-up survey to those who did not. Overall, most participants were single, aged between 18 and 20 years, identified as female and Anglo Australian, were employed, and earning between AUD$0 - $600 week. Most participants were not currently receiving psychological treatment or taking psychotropic medication. Table 2 summarises the clinical characteristics of the sample.

**Table 2:** Comparison analyses on outcomes from baseline to follow-up with expectation-maximisation algorithm applied

	Baseline		Follow-up		Comparison Statistic		Effect size
	(*n* = 70)		(*n* = 70)				
	*M*	*SD*	*M*	*SD*	*t* or *W*	*p*	*d* or *r*
HF MPS-45							
Self-oriented	78.73	13.77	72.06	13.85	*t =* 4.938	*p* < .001	*d* = −0.48
Socially-prescribed	56.80	14.76	51.19	12.89	*t =* 3.715	*p* < .001	*d* = 0.40
Other-oriented	53.76	12.62	51.31	12.90	*t =* 1.796	*p* = .077	*d* = 0.19
Rejection sensitivity	11.35	4.41	9.68	4.57	*t =* 5.412	*p* < .001	*d* = 0.37
BSI-53							
Depression (*Mdn*, *IQR*)	1.00	1.54	0.83	0.88	*W =* −3.739	*p* < .001	*r =* −0.47
Anxiety (*Mdn*, *IQR*)	0.83	1.67	0.62	0.88	*W =* −2.582	*p* = .010	*r =* −0.33
Hostility (*Mdn*, *IQR*)	0.60	1.00	0.29	0.60	*W =* −4.163	*p* < .001	*r =* 0.53
Perceived social support (*Mdn*, *IQR*)	5.79	1.58	5.92	1.32	*W =* −2.270	*p* = .023	*r =* −0.29
Objective social support	2.29	0.66	2.02	1.03	*t =* 2.287	*p* = .025	*d* = 0.31

HF MPS-45 = Hewitt and Flett Multidimensional Perfectionism Scale-45. BSI-53 = Brief Symptom Inventory-53. CERQ = Cognitive Emotion Regulation Questionnaire

A series of one-sample t-tests were conducted to compare the baseline characteristics of our sample to prior samples. These analyses revealed that socially-prescribed and self-oriented perfectionism were elevated in this sample compared to unrestricted university samples (*p’s* < .001; Hewitt & Flett, 1991). Self-oriented perfectionism was also elevated compared to psychiatric samples (*p* < .001) although socially-prescribed perfectionism was not (*p* = .437; Hewitt & Flett, 1991). In addition, levels of other-oriented perfectionism were consistent with means from an unrestricted university sample (*p* = .803) and a psychiatric sample in a previous study (*p* = .332; Hewitt & Flett, 1991). Moreover, the mean scores for depression, anxiety, and hostility were elevated compared to community samples (*p’s* < .001) but did not reach norms consistent with psychiatric samples (*p’s* < .001; Ryan, 2007). The mean scores for rejection sensitivity were also elevated compared to unrestricted university samples (*p* < .001; Ayduk et al., 2008), whilst perceived and actual social support were lower than means in unrestricted university samples (*p’s* =/< .040; Kazarian & McCabe, 1991; Reyes et al., 2020).

### Comparing Completers to Dropouts

As shown in Table 1, participants who dropped out had statistically higher levels of socially-prescribed perfectionism and were more likely to be unemployed than participants who completed the follow-up survey, although these differences were associated with trivial and small effect sizes, respectively. No other significant differences were evident between those who did and did not complete the follow-up survey.

## Effects of the Intentional Imperfection Program on Outcomes

As shown in Table 2, self-oriented and socially-prescribed perfectionism, rejection sensitivity, hostility, and depression and anxiety decreased from baseline to follow-up, while perceived social support increased from baseline to follow-up. Effect sizes for these changes ranged from just below the recommended minimum effect size representing a “practically” significant effect to moderate in magnitude. Objective social support evidenced a slight trivial decrease when analysing the total sample, but not when examining completers only (see Appendix Table 4).

## Analysis of the Feedback Survey

### Quantitative Questions

The quantitative analyses of the content, structure, readability, and use of practice techniques are summarised in Table 3. In terms of strengths, most participants reported that the program was: 1) very/quite understandable (96.8%), 2) very/quite easy to navigate (93.5%), 3) very/quite instructive (91.9%), 4) somewhat to very effective (87.1%), and 5) resulted in noticeable change (82.3%). Although most participants reported practicing program strategies for an average of 4.6 h in the two weeks following the completion of the program (87.1%), 30.65% of participants reported that they experienced barriers to completing the program and practicing the strategies. In regard to program weaknesses, 12.9% of participants reported the mode of program delivery was not or only a little effective, 61.3% reported the program would benefit from individualised feedback/guidance, and 61.3% reported the program was a bit too long (61.3%).

**Table 3:** Descriptive and frequency analyses on the quantitative data from the feedback survey

	*n*	*M*	%	*SD*
Understandability of content				
Somewhat understandable	2		3.2	
Quite understandable	17		27.4	
Very understandable	43		69.4	
Level of instruction				
A little instructive	2		3.2	
Somewhat instructive	3		4.8	
Quite instructive	26		41.9	
Very instructive	31		50.0	
Mode of delivery				
Not effective	1		1.6	
A little effective	7		11.3	
Somewhat effective	18		29.0	
Quite effective	23		37.1	
Very effective	13		21.0	
Ease of navigating the webpage				
Somewhat difficult	2		3.2	
Neither easy nor difficult	2		3.2	
Quite easy	26		41.9	
Very easy	32		51.6	
Length of resource				
Much too long	2		3.2	
A bit too long	38		61.3	
A good length	20		32.3	
Could have been a bit longer	1		1.6	
Could have been much longer	1		1.6	
Reported practicing the strategies	54		87.1	
Reported strategies practiced				
Mindful meditation	32		45.7	
Compassionate self-validation technique	41		58.6	
Distress tolerance	31		44.3	
Becoming more aware	26		37.1	
Social skills training	6		8.6	
Building better friendships	17		24.3	
Social goal-setting	19		27.1	
Hours spent practicing (*n* = 54)		4.63		2.93
Recommended individualised feedback/guidance	38		61.3	
Noticed changes after intervention	51		82.3	
Experienced barriers to treatment	19		30.6	

### Open-Ended Responses

The thematic analysis of the qualitative data resulted in four distinct themes and 10 sub-themes. Overall, these were related to strategies participants found most helpful (*helpful strategies*), changes participants noticed after completing the program (*changes noticed*), obstacles experienced during treatment (*barriers to treatment*), and recommendations to the delivery and structure of the program (*recommendations to the program*). Detailed reporting of the themes and sub-themes can be viewed in Appendix Table 5 and Appendix C although a summary of the findings is presented below.

Several participants found the mindfulness meditations most helpful (Theme 1) because they allowed them to become aware of their perfectionistic thoughts and behaviours and view them objectively and uncritically. Participants also identified the self-compassion strategy as helpful for overcoming self-criticism and for setting more realistic expectations of themselves. Other participants reported that the distress tolerance techniques were the most helpful, as they enabled them to engage with their negative emotions and realise that they could cope with them and deal with the problems from which they arose. Others reported that the social skills training was the most useful as it was effective in improving their perspective taking and confidence in reaching out to others to build better social connections. Thus, it seems that the multifaceted approach of the IIP may address people’s various needs and preferences.

In terms of the changes noticed after completing the program (Theme 2), the majority reported improvements, particularly in relation to having more realistic expectations and greater respect for themselves and others, as well as having a better understanding of their perfectionistic tendencies and the consequences of them. Many also reported having more self-compassion and improved social interactions because of the program. Nineteen participants reported barriers to treatment (Theme 3) that hindered them from completing the program and/or practicing the strategies. These hindrances included a lack of time or opportunity to practice the strategies (e.g., COVID restrictions prevented practicing social skill strategies with friends, unable to fit in with work and university commitments), embarrassment and reluctance to think of themselves as perfectionistic, and difficulty focusing on the program’s content due to the length of the program.

Finally, in terms of recommendations for program improvement (Theme 4), the most common was to make the program individualised so that participants could choose the components most relevant to their perfectionistic thoughts and behaviours based on their responses to the baseline assessment. Finally, some suggested expanding the program to include a greater number of strategies to help them manage their perfectionism and challenge their perfectionistic thinking.

## Discussion

The primary aim of this study was to assess the acceptability and potential effectiveness of the IIP in reducing the consequences of perfectionism among university students to determine whether a randomised controlled trial is warranted. Consistent with predictions, engagement with the IIP was associated with decreases in rejection sensitivity, hostility, depression, anxiety, and increases in perceived social support. Contrary to predictions, significant changes in objective social support did not occur from baseline to follow-up. Although not specifically targeted by the intervention, self-oriented and socially-prescribed perfectionism decreased from baseline to follow-up. The quantitative and qualitative analyses of the feedback survey indicated that the program was generally well accepted, and also highlighted areas for improvement, that for the most part, could easily be resolved. Together, these findings provide preliminary evidence for the usefulness of the IIP in helping students to manage perfectionism.

The magnitude of change in depression and anxiety observed in this study is comparable to other pre-post changes in psychological distress achieved by much longer interventions targeting perfectionism (ranging from 8 to 12 sessions). For example, two previous meta-analyses examining the efficacy of cognitive-behavioural therapy treatment for perfectionism reported moderate decreases in symptoms of anxiety and depression (Lloyd et al., 2015; Suh et al., 2019). However, given that this was a feasibility study that did not directly compare outcomes to a longer intervention, further research is needed to confirm this.

Despite its preliminary efficacy, the IIP appeared to be less effective in increasing objective social support than it was for increasing perceived social support. A possible explanation may be that participants were required to engage in physical distancing measures to prevent the spread of COVID-19 while participating in this study, and therefore, had limited opportunities to practice the recommended strategies in the two-week period. As previous research suggests that increased perceived social support, rather than actual social support, predicts better mental health outcomes (Eagle et al., 2019), the program may not need to improve objective indices of social support. Nevertheless, further research is needed to clarify if the IIP can increase objective social support during a non-pandemic context.

Interestingly, we found that completion of the IIP yielded changes on two facets of perfectionism, which was not predicted by the model. The PSDM states that perfectionism is a stable trait and that reductions in perfectionism may require longer and more intensive treatments (Hewitt et al., 2015). Studies that have successfully reduced some forms of perfectionism identified socially-prescribed perfection to be particularly resistant to treatment (Glover et al., 2007). However, our findings showed that the IIP was associated with small reductions in socially-prescribed and self-oriented perfectionism. Therefore, changes in other-oriented perfectionism may be possible if future iterations of the IIP devote more attention to it. In the current version, only the interpersonal hostility videos specifically targeted problematic behaviours/cognitions associated with other-oriented perfectionism.

The qualitative findings suggest that participants adhered to homework tasks, enjoyed the program, and found it useful. In particular, participants reported that the mindfulness meditations, self-compassion strategies, distress tolerance activities, and social skills training were most beneficial to them. However, the qualitative responses also indicted that a few changes to the program may help. Although the IIP is much shorter than existing perfectionism treatments, more than half of participants reported that the program was too long. As some participants reported that they preferred to complete the program over a longer time-frame, it may not be that the program needs to be shorter, but that participants need to be given flexibility as to when they complete the modules. Many participants also reported that they would have benefited from individualised feedback and/or guidance. Refinements to the IIP could include providing participants with feedback on their baseline assessment, which directs them to specific modules that may be particularly helpful to them.

## Limitations

A number of limitations must also be considered when drawing conclusions from the current study. First and foremost, as we did not reach our required sample size indicated by our a priori power analysis, we were not able to test the validity of the PSDM tenets. Thus, this paper only presents on treatment outcome. Second, given the absence of a control group, the findings of the study cannot be confidently attributed to the intervention, and as such, the observed outcomes should be considered with caution. Third, our comparison analyses showed that those who dropped out were more likely to have higher levels of socially prescribed perfectionism and were more likely to be unemployed. Our sample also predominately consisted of Anglo Australian females. As such, our sampling procedure may have limited the generalisability of our results to a specific subset of those with perfectionism. Lastly, we were also unable to observe participants’ completion of the modules and time spent on the website and had to rely to self-report. To address these limitations, a future randomised controlled trial would benefit from obtaining a larger, more diverse sample to increase statistical power, using software that objectively measures program engagement and homework compliance to reduce reporting. Additionally, the study would benefit from ensuring the IIP is culturally appropriate and retains participants with higher levels of socially-prescribed perfectionism to increase the generalisability of our results.

## Conclusions

Several studies have shown that both perfectionism and psychological distress are highly prevalent among university students, and barriers such as time and financial constraints means that most do not seek or receive any psychological treatment (Lipson et al., 2019). Our feasibility study showed preliminary support for the the use of a theory-driven approach for targeting multidimensional perfectionism using the IIP. The IIP may be a practical alternative to longer, more intensive treatments for university students as it is brief, affordable, and can easily be disseminated. The next step will be to revise the program based on the current data and to test the IIP in a randomised controlled trial.

## Data Availability

The data that support the findings of this study are available from the corresponding author, Melissa M. Norberg, upon reasonable request.

## Appendix A

Prior to beginning the learning modules, participants were shown an introductory video (1:21 min) which outlined the program’s content and the types of skills that would be taught. Participants were asked to download the program worksheet and were told that they would be prompted to use the worksheet to answer questions during the program.

In the first learning module, participants were provided psychoeducation via a brief video (6:02 min) describing perfectionism, the various types of perfectionism, and the Perfectionism Social Disconnection Model. Following this video, participants were prompted to reflect on their own experience of perfectionism and its impact on their daily life, social functioning, and mental health using their worksheet.

The second learning module consisted of a video (4:45 min) that provided psychoeducation on interpersonal sensitivity, examples of interpersonally sensitive behaviour, and outlined skills to manage interpersonally sensitive behaviour (i.e., mindfulness, compassionate self-validation, and distress tolerance; Berenson et al., 2016; Joss et al., 2020; Keng & Tan, 2018; Sakiz & Saricam, 2015). Participants were then prompted to reflect on their own experiences of interpersonally sensitive behaviour and its impact on their relationships using their worksheet. Afterwards, participants were shown a video (5:18 min) providing psychoeducation on the benefits of mindfulness in managing and reducing rejection sensitivity (Joss et al., 2020; Keng & Tan, 2018). Participants were then shown a mindful meditation video (11:11 min) and were prompted to reflect on their experience with the mindful meditation and its utility using their worksheet. Next, participants were shown a video (4:37 min) explaining emotional regulation and its relationship with perfectionism. The video also contained explicit training on how to regulate emotions using self-compassion to adopt a mindset of care and empathy for oneself. Participants were then prompted to reflect on this activity in their worksheet. Lastly, participants were provided with psychoeducation via a video (4:37 min) on distress tolerance and increasing distress tolerance. Participants then reflected on their own capacity to tolerate distress and strategies to increase this skill using their worksheet.

The third learning module provided psychoeducation via video (3:46 min) on interpersonal hostility, examples of hostile behaviour, and outlined skills to manage hostile behaviours (i.e., experiential ownership and social skills training; Gresham, 1985; Heppner et al., 2008; Hofmann et al., 2011; Spence, 2003; Zaragoza et al., 1991). Participants were then encouraged to reflect on their own experience of interpersonal hostility using their worksheet. Afterwards, participants were shown a video (4:15 min) guiding them through an activity that encouraged participants to increase their awareness of their own and others’ emotions by reflecting on a social situation in which the participant responded with hostility. Participants were then asked to reflect on this experience and its implications using their worksheet. Next, participants were shown a video (0:40 min) which briefly discussed some interpersonal hostile behaviours (i.e., cool disposition, non-disclosure and distancing oneself from social situations) and its link to perfectionism. Participants were then shown a series of scenarios via different videos which included examples of common perfectionistic behaviours demonstrated by actors. These scenarios included a student reacting negatively to receiving feedback from an instructor (5:48 min), an individual perceiving rejection from a peer (3:23 min), and an individual displaying hostile behaviour in a group setting (10:16 min). After each video, participants were shown the same scenarios again via video (9:15 min), but with the individual demonstrating adaptive responses instead. Participants were then asked to identify the behaviours in each interaction that were unhelpful and helpful using their worksheet.

The fourth learning module was related to social disconnection. Participants were shown a video (1:27 min) which explained subjective and objective social disconnection, its relationship to perfectionism, and outlined skills to increase social connectedness (i.e., friendship enrichment activities; Kim & Kim, 2011; Stevens, 2001). Participants were then prompted to use their worksheet to consider their own level of social connectedness. Next, participants were shown a video (5:40 min) guiding them through an activity called ‘Building Better Friendships’ which encouraged participants to think about themselves as friends and contemplate the qualities of a good friend. This activity, originating from Leahy et al.’s (2011) idea of needing to support others to receive support, explained how domineering interpersonal styles can lead to one-sided friendships. Participants then reflected on the importance of mutual support and implementation of the technique using their worksheet. Next, a video (3:27 min) guided participants through an activity to set friendship goals. In this activity, participants are asked to map out their social network and reflect on their available support networks. Participants also were encouraged to identify individuals they may want to cultivate stronger bonds with and set social goals for improving their connections with others using their worksheets.

The fifth learning module summarised the content in the program and provided participants with a homework worksheet to practice the skills taught during the IIP by integrating its teachings into their everyday lives. To help with this, participants were asked to map out their schedule for the next two weeks and block out time for daily practice. Participants were encouraged to again reflect on the impact that perfectionism has had on their life, but this time, they were to identify strategies that may help them to reduce its impact. Next, participants were shown a final video (4:39 min) which informed them that they would be contacted in two weeks to complete a feedback survey. The video also thanked participants for their participation and provided them with resources to access additional mental health services if needed.

References

Berenson, K.R., Gregory, W.E., Glaser, E. (2016)*.* Impulsivity, rejection sensitivity, and reactions to stressors in borderline personality disorder. *Cognitive Therapy and Research, 40*(4), 510–521. 10.1007/s10608-015-9752-y

Gresham, F. M. (1985). Utility of cognitive-behavioural procedures for social skills training with children: A critical review. *Journal of Abnormal Child Psychology,* 13(3), 411–423. 10.1007/BF00912725

Heppner, W. L., Kernis, M. H., Lakey, C. E., Campbell, W. K., Goldman, B. M., Davis, P. J., & Cascio, E. V. (2008). Mindfulness as a means of reducing aggressive behavior: Dispositional and situational evidence. *Aggressive Behavior: Official Journal of the International Society for Research on Aggression, 34*(5), 486–496. 10.1002/ab.20258

Hofmann, S. G., Grossman, P., & Hinton, D. E. (2011). Loving-kindness and compassion meditation: Potential for psychological interventions. *Clinical Psychology Review, 31*(7), 1126–1132. 10.1016/j.cpr.2011.07.003

Joss, D., Lazar, S.W. & Teicher, M.H. (2020). Nonattachment predicts empathy, rejection sensitivity, and symptom reduction after a mindfulness-based intervention among young adults with a history of childhood maltreatment. *Mindfulness*, *11*, 975–990. 10.1007/s12671-020-01322-9

Keng, S. L., & Tan, H. H. (2018). Effects of brief mindfulness and loving-kindness meditation inductions on emotional and behavioral responses to social rejection among individuals with high borderline personality traits. *Behaviour Research and Therapy, 100*, 44–53. 10.1016/j.brat.2017.11.005

Kim, H. U., & Kim, K. W. (2011). Influence of friendship to academic persistence and drop out and mediation effect of school adaptation. *Journal of Fashion Business*, *15*(4), 87–109.

Leahy, R. L., Tirch, D., & Napolitano, L. A. (2011). *Emotion Regulation in Psychotherapy: A Practitioner’s Guide.* Guilford Publications.

Sakiz, H., & Saricam, H. (2015). Self-compassion and forgiveness: The protective approach against rejection sensitivity. *International Journal of Human and Behavioral Science*, *1*(2), 10–21. 10.19148/ijhbs.58217

Spence, S. H. (2003). Social skills training with children and young people: Theory, evidence, and practice. *Child and Adolescent Mental Health, 8*(2), 84–96. 10.1111/1475-3588.00051

Stevens, N. (2001). Combating loneliness: a friendship enrichment programme for older women. *Ageing & Society, 21*(2), 183–202. 10.1017/s0144686x01008108

Zaragoza, N., Vaughn, S., & Mcintosh, R. (1991). Social skills interventions and children with behavior problems: A review. *Behavioral Disorders, 16*(4), 260–275.

## Appendix B

Table 4

**Table 4:** Comparison analyses on outcomes from baseline to follow-up on the completer sample

	Baseline		Follow-up		Comparison Statistics		Effect size
	(*n* = 62)		(*n* = 62)				
	*M*	*SD*	*M*	*SD*	*t* or *Z*	*p*	*r* or *d*
HF MPS-45							
Self-oriented	79.52	13.59	71.73	14.69	*t =* 5.862	*p* = .001	*d =* 0.55
Socially-prescribed	55.40	14.21	51.63	13.65	*t =* 2.677	*p* = .010	*d =* 0.27
Other-oriented	54.08	12.37	52.37	13.35	*t =* 1.250	*p* = .216	*d =* 0.13
Rejection sensitivity	11.28	4.57	9.53	4.85	*t =* 5.472	*p* < .001	*d =* 0.37
BSI-53							
Depression (*Mdn*, *IQR*)	1.00	1.37	0.83	1.04	*Z =* −3.287	*p* = .001	*r =* −0.42
Anxiety (*Mdn*, *IQR*)	0.83	1.67	0.50	1.04	*Z =* −2.181	*p* = .029	*r =* −0.28
Hostility (*Mdn*, *IQR*)	0.60	1.00	0.40	0.80	*Z =* −3.545	*p* < .001	*r =* −0.45
Perceived social support (*Mdn*, *IQR*)	5.87	1.27	6.00	1.17	*Z =* −2.691	*p* = .007	*r =* −0.34
Objective social support	2.27	0.68	2.28	0.78	*t =* −0.184	*p* = .854	*d =* 0.01

HF MPS-45 = Hewitt and Flett Multidimensional Perfectionism Scale-45. BSI-53 = Brief Symptom Inventory-53. CERQ = Cognitive Emotion Regulation Questionnaire

## Appendix C

The thematic analysis of the qualitative data resulted in four distinct themes and 10 sub-themes (see table below). These were related to strategies participants found most helpful (*helpful strategies*), changes participants noticed after completing the program (*changes noticed*), obstacles during treatment (*barriers to treatment*), and recommendations to the delivery and structure of the program (*recommendations to the program*).

Table 5 

**Table 5:** Themes and sub-themes from the qualitative data analysis from the feedback survey. Themes and sub-themes from the qualitative data analysis from the feedback survey

Themes	Sub-themes	Examples of codes
*Helpful strategies*	Mindfulness	Awareness, relaxation, reduced impulsivity
	Self-compassion	Positive mindset, self-compassion
	Distress tolerance	Engagement, acceptance
	Social skills	Understanding, social relatedness, interpersonal skills
*Changes noticed*	Towards self	Reconsideration of achievement, understanding, self-compassion
	Towards others	Reconsideration of expectations, awareness, compassion
*Barriers to treatment*	Internal barriers	Uncomfortable feelings, difficult to stay focused
	External barriers	Lack of opportunity, conflicting commitments, significant life events
*Recommendations to the program*	Recommended removing	Strategies in the program, structure of the program
	Recommended adding	Other techniques and resources to manage perfectionism

### Helpful Strategies

Several participants reported that the mindfulness strategies in the program were most helpful to them. Some participants reported that the mindful meditation helped them to become more aware of ‘*feelings and sensations’*, ‘*where* [their] *perfectionism creeps into my everyday life’* and *‘the perspective of others’.* Some participants also reported that engaging in mindful meditation made them feel more relaxed. For instance, ‘*it calms me down and most importantly, it eases my mind, in the end, I’m not fighting my thoughts anymore, I’ve learnt how to let them go (most of the time)’.*

Several participants also reported benefits from the self-compassion strategies taught in the program. Some participants reported that these strategies helped them to adopt a ‘*positive mindset’* which ‘*reminded them to set reasonable expectations for themselves’* and made them feel *‘better and more capable of managing things.’* Many participants also reported that the self-compassion strategies allowed them to be less ‘*critical*’, ‘*harsh*’ or ‘*judgemental*’ towards themselves.

A few participants also reported that distress tolerance strategies were the most helpful of the program. Some participants reported that the distress tolerance strategies helped them to engage with their negative emotions. For example, ‘*I usually ‘bury’ these kinds of feelings to get on with it. It was a challenge, but I physically felt I was able to engage with my emotions’.* Other participants reported being able to ‘*accept the distress’* associated with their perfectionism (e.g., ‘*helped me realised that I am capable of dealing with problems that I encounter daily’* and *‘learning I am okay even if it is not ‘perfect’).*

A few participants reported that the activities on social skills in the program were most helpful to them as it helped them to understand the thoughts of others. For instance, *‘learning to understand how my friends were thinking and seeing from their point of view. It made me feel like I was closer to them and that it was easier to have conversations.’* Other participants also reported that the activities helped them to ‘*reach out to the friends I* [they] *already have to become closer and make new friends*’ and improve their interpersonal skills such as taking the time to *‘really listen to others, thank them for their time and ask them questions’.*

### Changes Noticed

A large majority of participants reported noticing changes after completing the program. The treatment appeared to have had a positive effect on participants’ well-being as several responses involved noticing changes in themselves. Some participants reported reconsidering their standard of perfection: *‘my expectations for myself and other people are not as strong, I treat myself with more respect and therefore, I also treat other individuals better.’* Some participants also reported greater understanding of thoughts and feelings related to perfectionism: ‘*I also have a better understanding of how my perfectionistic tendencies manifest and why’*. Several participants also reported being more self-compassionate, ‘*kinder’, ‘understanding’* and *‘patient’* towards themselves giving themselves ‘*the benefit of the doubt and also that room to breathe and relax without feeling guilty*.’

Several participants also noticed changes towards their interactions with others and reconsidering their expectations of them: *‘I have also reflected and identified behaviours or situations where I was unfair or had unfair expectations on others due to perfectionism and have been able to start addressing them’.* Some participants reported ‘*I am more aware of how I treat my friends’*, ‘*more aware of asking other people what is happening in their lives’* and ‘*have become more aware that I often talk about myself and should work on becoming a better listener for my friends’*. As a result, many participants reported being more ‘*compassionate*’, ‘*mindful*’, ‘*patient*’, ‘*understanding*’, ‘*respectful*’ and ‘*considerate*’ towards others.

### Barriers to Treatment

Some participants reported barriers which hindered them from completing the program and/or practicing the strategies. A few participants discussed internal barriers to treatment such as uncomfortable feelings arising from activities which involved participants reflecting on their perfectionism. For example, participants reported ‘*negative feelings coming up’*, ‘*I felt embarrassed [sic] to think of myself as a perfectionist’* and a ‘*reluctance to accept’*. Some participants also reported that it was *‘difficult to stay focused’* which some attributed to the length of the program; ‘*it was too hard to complete in one go’*, and ‘*it was too long that I became disengaged.*’

Some participants also discussed external barriers to treatment such as a lack of opportunity to practice the strategies as they *‘did not have many opportunities to interact with others’* and ‘*did not have any university work or assignments during this period*’. On the other hand, some participants also reported ‘*difficulties in juggling commitments* (*university, work, volunteering, family*)’ which prevented them from ‘*setting time aside*’ for the program. A few participants also reported recent significant life events (e.g., ‘*familial loss*, ‘*extremely traumatic event’*) which disrupted their engagement with the program and practice of the strategies.

### Recommendations to the Program

Although many participants reported that they would not recommend changing the program, some participants recommended removing components from the program. For instance, some participants suggested removing specific strategies and/or techniques such as the mindful meditation, compassionate self-validation and social skills as they felt these strategies were ‘*irrelevant’* or ‘*did not work*’ for their perfectionism. However, consistent with the data reported above on the benefits others gained from these strategies/techniques, many of these participants also acknowledged that these strategies may be favourable to other participants and thus should still be included. For example, *‘I think they are all helpful in their own way and some of the different resources may benefit some individuals more than others.’* Other participants reported that they would prefer a more individualised program to suit the perfectionism that they experienced, such as making a *‘quiz at the beginning to direct the user to the part of perfectionism that is most relevant to them’.*

**S**ome participants also recommended adding components to the program that they would find useful in managing their perfectionism. These included: ‘*thought challenging which is something that has helped me significantly…I think the structure of thought challenging is very helpful’,* or *‘communication, ability to say no, conflict solving’* and ‘*a sports section, as being an athlete managing my perfectionism can be quite difficult during training sessions an* [sic] *racing’.* Other participants recommended adding ‘*other resources that could help with perfectionism (Smiling Mind for example)’*.
